# Comparison of chikungunya viruses generated using infectious clone or the Infectious Subgenomic Amplicons (ISA) method in *Aedes* mosquitoes

**DOI:** 10.1371/journal.pone.0199494

**Published:** 2018-06-28

**Authors:** Souand Mohamed Ali, Anubis Vega-Rúa, Jean-Sélim Driouich, Xavier de Lamballerie, Anna-Bella Failloux, Antoine Nougairède

**Affiliations:** 1 Unité des Virus Émergents (UVE: Aix-Marseille Univ–IRD 190 –Inserm 1207 –IHU Méditerranée Infection), Marseille, France; 2 Department of Virology, Arboviruses and Insect Vectors, Institut Pasteur, Paris, France; Singapore Immunology Network, SINGAPORE

## Abstract

Reverse genetics systems provide the opportunity to manipulate viral genomes and have been widely used to study RNA viruses and to develop new antiviral compounds and vaccine strategies. The recently described method called ISA (Infectious Subgenomic Amplicons) gives the possibility to rescue RNA viruses in days. We demonstrated in cell culture that the use of the ISA method led to a higher genetic diversity of viral populations than that observed using infectious clone technology. However, no replicative fitness difference was observed. In the present study, we used the chikungunya virus as a model to compare in *Aedes aegypti* and *Aedes albopictus* mosquitoes the genotypic and phenotypic characteristics of viruses produced either from an infectious clone or using the ISA method. We confirmed the results found *in cellulo* corroborating that the use of the ISA method was associated with higher genetic diversity of viral populations in mosquitoes but did not affect the vector competence validating its use for *in vivo* experiments.

## Introduction

Reverse genetics systems that give the possibility to rescue infectious viruses from DNA copies of their genomes, are important tools to explore viral life cycle and to contribute to the development of new antiviral compounds and vaccine candidates [1–4].

Numerous reverse genetics systems that allow producing wild type and genetically modified viruses have been previously developed, each associated to specific benefits and limitations [1]. Infectious clones are widely used but commonly difficult to create, in particular because of the instability and toxicity of some viral sequences into bacteria [5–7]. To overcome these difficulties, bacterium-free approaches alternative methods have been developed, such as the ISA (infectious subgenomic amplicons) method that was recently applied to a large panel of single-stranded positive-sense RNA viruses [6, 8–10].

Using the Chikungunya virus (CHIKV; family *Togaviridae*; genus *Alphavirus*) as a model, we previously demonstrated *in cellulo* that the use of the ISA method generated a higher genetic diversity of viral populations than that observed with the use of an infectious clone [11]. We concluded that PCR-based reverse genetics methods -such as the ISA method- are associated with artificial viral heterogeneity because the polymerases employed during PCR amplification generate a number of undesired nucleotide changes. Since the structure of viral populations can shape virus phenotype, we previously investigated the impact of using the ISA method on viral phenotype *in cellulo*, in comparison with using an infectious clone [12–14]. We did not identify any difference in the replicative fitness of the viruses produced by both methods [11].

Accordingly, the main objective of the current study was to compare *in vivo* the impact on genotype and phenotype of using either the ISA method or an infectious clone. As in our previous work, we used CHIKV as a model virus and investigated the genotypic and phenotypic characteristics of viruses produced by both methods in the two main mosquito vectors of CHIKV, *Aedes aegypti* and *Aedes albopictus*.

## Results and discussion

### Production of viruses

A previously described infectious clone of the LR2006 strain of CHIKV was used (i) to transfect cells and generate infectious particles (named IC_virus) and (ii) to produce subgenomic amplicons used during the ISA procedure to rescue infectious virus (named ISA_virus) [15]. Infectious clone and subgenomic amplicons were transfected into HEK-293 cells. Viruses were passaged twice in Vero cells. Virus stocks were used to infect mosquitoes.

### Mosquito oral infections

*Ae*. *aegypti* and *Ae*. *albopictus* females were orally infected with an infectious blood meal (final infectious titre: 10^6.8^ TCID_50_/mL). After removing non-engorged females, mosquitoes were incubated 6/9 days. For each experimental condition, a group of 30 mosquitoes was used to assess vector competence.

### Evaluation of genetic diversity in virus stocks and *Aedes* mosquitoes

The genetic diversity was evaluated using complete viral genome sequences from virus stocks used to infect mosquitoes and from positive mosquito heads (presence of virus was assessed using a TCID_50_ assay; see below). Data were generated by next generation sequencing and we considered in our analyses all the substitutions with a frequency ≥2%.

We first evaluated the genetic diversity in virus stocks used to infect mosquitoes (the sequence of the infectious clone was used as a reference). Not surprisingly, transfection of the infectious clone led to production of clonal viral population and no mutation was detected in the IC_virus. In contrast, transfection of amplicons led to a higher genetic diversity and 13 mutations were detected in ISA_virus (**Table 1**). This result is in accordance with our previous work [11]. Of note, only minority mutations (substitution frequency <50%) were detected in ISA_virus and therefore both viruses had identical consensus sequence.

**Table 1:** Subtstitutions generated using the ISA method and detected in *Aedes* mosquitoes infected by the ISA_virus.

Nucleotide position	Original substitution frequency*	Region	nucleotide change	aa change	Number of mosquitoes with the substitution detected on viral genome		
					*A*. *aegypti* 6 dpi (n = 8)	*A*. *aegypti* 9 dpi (n = 6)	*A*. *albopictus* 6 dpi (n = 5)
502	0,21	nsP1	A→G	-	1	0	1
710	0,03	nsP1	T→C	-	0	0	0
881	0,03	nsP1	A→C	K→Q	0	0	0
1105	0,03	nsP1	C→A	-	0	0	0
1590	0,22	nsP1	T→G	L→R	1	0	1
2458	0,28	nsP2	T→C	-	4	0	2
6923	0,23	nsP4	A→G	T→A	3	2	4
7628	0,28	C	C→T	P→L	4	0	2
8612	0,02	E2	A→G	E→G	0	0	0
8766	0,15	E2	A→G	-	4	0	2
9100	0,1	E2	A→G	N→D	0	3	1
9690	0,02	E2	T→C	-	0	0	0
11644	0,1	3'UTR	A→G	-	0	3	1

List of the substitutions detected in virus stock (ISA_virus) used to infect *Aedes* mosquitoes. None of these mutations were detected in mosquitoes infected by the IC_virus. “dpi” means days post-infection.

* frequency found in virus stock.

**Fig 1:**
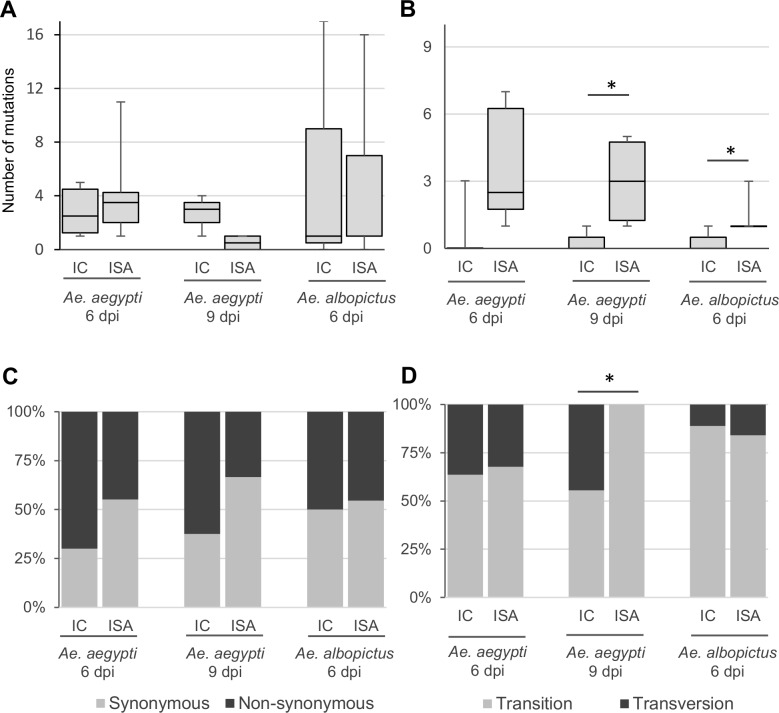
Genetic diversity in *Aedes* mosquitoes. Panels **A** and **B** represent the number of minority and majority mutations respectively detected in mosquito heads (substitution frequency < and ≥ 50% respectively). Mutation characteristics are represented in panels **C** (Non synonymous; Synonymous mutations) and **D** (Transition; Transversion). In Panels **A** and **B**, the bottom and top of the box represent first and third quartiles, the band inside the box represents median value and the ends of the error bars represent minimum and maximum values. IC and ISA mean group of mosquitoes infected by IC_virus and ISA_virus respectively. “dpi” means days post-infection. * means that a significant difference (*p* value <0.05) was observed between mosquitoes infected by IC_virus and ISA_virus.

We then evaluated the genetic diversity from grounded heads of *Aedes* mosquito. Based on our experiment, it is difficult to generate complete genome sequences from individual mosquito heads. Thereby, we initiated analyses with eight heads per condition. Finally, data from three to eight heads per condition were used. To evaluate the impact of the reverse genetics method used, we compared the number of minority and majority mutations (substitution frequency < and ≥ 50% respectively) found for each condition. No clear trend can be found with minority mutations (**Fig 1A**): no impact of the reverse genetic method was observed in *Ae*. *aegypti* and *Ae*. *albopictus* heads at day 6 post-infection and the ISA method was associated with a slightly (not significant) lower number of mutations at day 9 post-infection in *Ae*. *aegypti*. Rather, the ISA method was associated with a higher number of majority mutations in all conditions (**Fig 1B**). This difference was significant in *Ae*. *aegypti* mosquitoes (Wilcoxon test; *p* value = 0.012 and 0.047 at 6 and 9 days post-infection, respectively). These findings suggested that in mosquito infected by ISA_virus, some of minority variants present in the blood meals were probably selected during the passage of the midgut barrier. To confirm this hypothesis, we specifically followed the mutations detected on ISA_virus stock (**Table 1**). We observed that none of the mutation initially present with a frequency <10% were found in mosquitoes. In contrast, all the mutations initially present with a frequency ≥10% were found in at least 2 mosquitoes (ranging between 2 and 9). Most of them were majority mutations in mosquitoes (21/39 of which 21 were totally fixed). Altogether, these findings demonstrated that genetic diversity in mosquitoes was higher when the ISA method was used to produce the virus.

In all conditions, the proportion of transition ranged between 56% and 100% (**Fig 1C**). The ISA method was associated with a significant higher number of transition at day 9 post-infection in *Ae*. *aegypti* (Fischer test; *p* value = 0.005). The reasons for this discrepancy remained unclear. No significant difference in the proportion of synonymous and non-synonymous mutations was found (**Fig 1D**). In all conditions, mutations detected in mosquitoes were distributed throughout the whole genome (**Fig A in S1 File**).

### Evaluation of vector competence in *Aedes* mosquitoes

**Fig 2:**
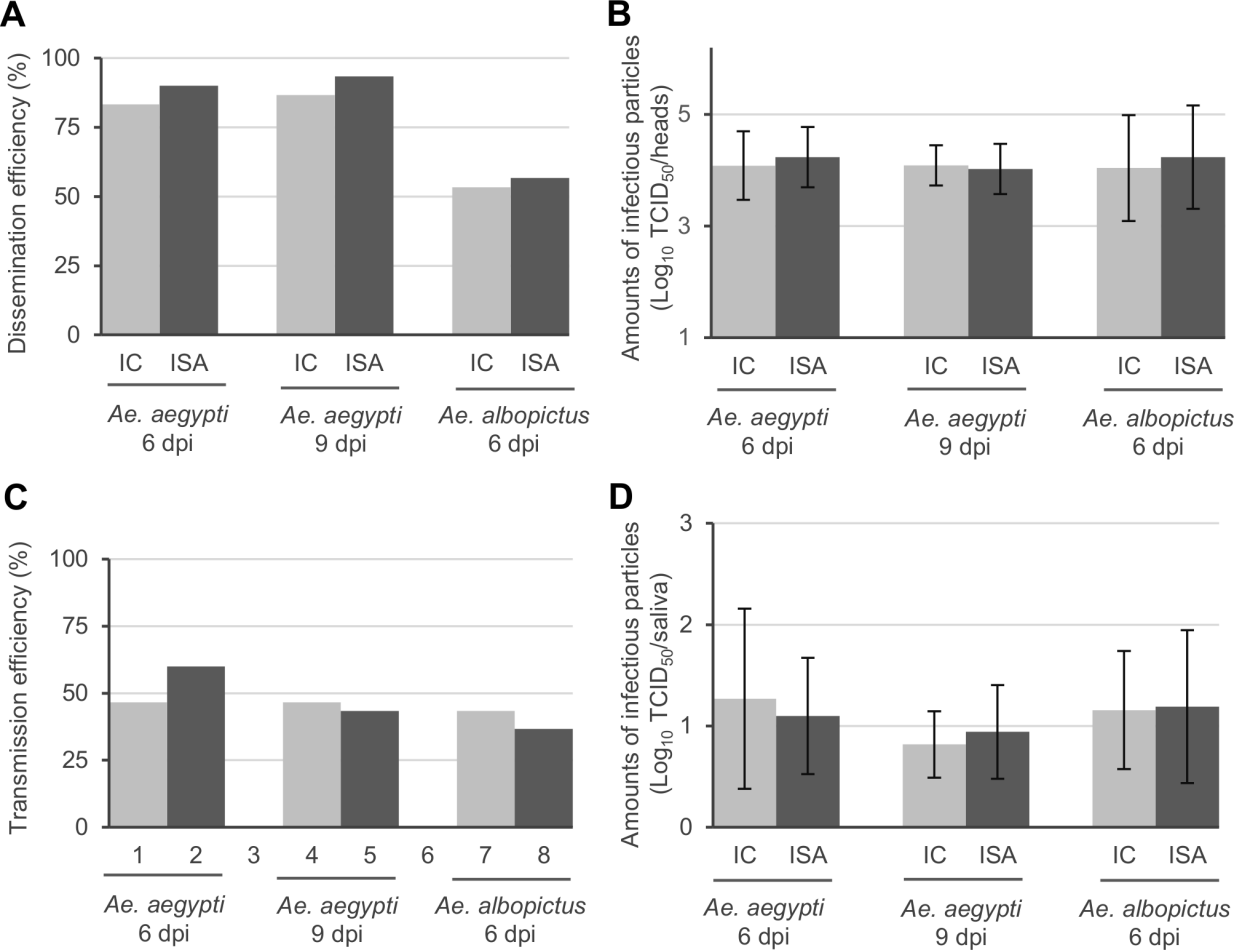
Vector competence in *Aedes* mosquitoes. Dissemination efficiency (DE; proportion of mosquito with infectious virus in the head) was determined by testing mosquito heads with a TCID_50_ assay (Panel **A**). The intensity of viral dissemination was assessed by comparing amounts of infectious particles in collected heads (Panel **B**; only positive heads were taken into account). Transmission efficiency (TE; proportion of mosquitoes with infectious virus in the saliva) was determined by testing mosquito saliva with a TCID_50_ assay (Panel **C**). The intensity of viral transmission was assessed by comparing amounts of infectious particles in collected saliva (Panel **D**; only positive saliva were taken into account). In panels **B** and **D**, the average values are shown and error bars represent standard deviation. IC and ISA mean group of mosquitoes infected by IC_virus and ISA_virus respectively. “dpi” means days post-infection.

To assess the ability of *Ae*. *aegypti* and *Ae*. *albopictus* mosquitoes to allow IC_virus and ISA_virus to pass through the midgut barrier, dissemination efficiency (DE; proportion of mosquitoes with infectious virus in the head) was determined by testing individual grounded heads with a TCID_50_ assay (**Fig 2A**). Similar DE values ranging from 83 to 93% were observed with *Ae*. *aegypti* at days 6 and 9 post-infection for both viruses. Similarly, close DE values ranging from 53 to 57% were found with *Ae*. *albopictus* at day 6 post-infection. The intensity of viral dissemination was also assessed by comparing amounts of infectious particles in collected heads (only positive heads were taken into account). In all conditions, mean values were similar ranging from 4.0 to 4.2 log_10_ TCID50/head (**Fig 2B**). All these findings indicated that the virus dissemination in *Aedes* mosquitoes was not significantly impacted by the reverse genetic method used to generate the virus.

To determine the ability of *Ae*. *aegypti* and *Ae*. *albopictus* mosquitoes to sustain transmission of IC_virus and ISA_virus, transmission efficiency (TE; proportion of mosquito with infectious virus in the saliva) was assessed by testing individual collected saliva with a TCID_50_ assay (**Fig 2C**). TE values ranging from 37 to 47% were found in all cases except with *Ae*. *aegypti* infected with the ISA_virus at day 6 post-infection (60%). However, this difference was not significant. The intensity of viral transmission was also assessed by comparing amounts of infectious particles in collected saliva (only positive heads were taken into account). In all conditions, mean values were similar ranging from 0.8 to 1.3 log_10_ TCID50/saliva (**Fig 2D**). All these findings indicated that the virus transmission was not affected by the reverse genetic method used to generate the virus.

## Conclusion

The ISA method represents a technological breakthrough for the production of recombinant RNA viruses [10]. We previously demonstrated that the ISA method conducted to higher genetic diversity of viral populations *in cellulo* but this genotypic difference was associated with no significant difference in replicative fitness [11]. In this study, we compared in *Aedes* mosquitoes phenotypic and genotypic characteristics of CHIKVs produced using ISA- and IC-based reverse genetics methods. We observed a higher genetic diversity of viral population in heads of mosquitoes infected by viruses generated using the ISA method. However, we found that vector competence was not affected by this genotypic difference since it was identical regardless the reverse genetic procedure used validating the use of both methods for *in vivo* experiments. These findings suggest that a certain level of artificial heterogeneity in virus population is easily supported by CHIKV during its replication in *Aedes* mosquitoes highlighting the high level of mutational robustness of this RNA virus. However, it is also possible that deleterious mutants were already negatively selected during the first round of viral replication in cell culture. This hypothesis remains to be tested by analysing the early evolutionary events that occurred *in cellulo*.

## Methods

### Cells

Vero cells (ATCC number CCL-81) were maintained at 37°C/5 % CO_2_ in minimal essential medium (Life Technologies) with 7 % heat-inactivated foetal bovine serum (FBS; Life Technologies), 1 % penicillin/streptomycin (PS; 5000 U.ml^−1^ and 5000 μg.ml^−1^; Life Technologies) and 1% Glutamine (Gln; 200 mmol.l^−1^; Life Technologies). HEK-293 cells (ATCC number CCL-1573) were maintained at 37°C/ 5 % CO_2_ in the same medium supplemented with 1 % non-essential amino acids (Life Technologies).

### Mosquitoes

We used in this study (i) the PAEA colony of *Ae*. *Aegypti* established from a population collected as larvae in urban settings of French Polynesia in 1994 and (ii) the BP strain of *Ae*. *Albopictus* collected as larvae in urban settings of Binh Phuoc (Vietnam) in 2011 and maintained in laboratory for 9 generation before experimental infections. After egg hatching, larvae were split in pans filled with one liter tap water supplemented with yeast tablets. Emerging adults were maintained at 28°C, 80% relative humidity in cages with a 14h:10h light:dark cycle and supplied ad libitum with a 10% sucrose solution.

### CHIK infectious clone

We used a previously described CHIKV infectious clone in this study (LR2006 strain of CHIKV [15]; GenBank accession EU224268).

### Preparation of DNA fragments for the ISA procedure

DNA fragments for the ISA procedure were prepared as previously described [11]. Briefly, the complete viral genome was amplified by PCR, using the infectious clone as template, in three overlapping DNA fragments. The 5’-extremity of the first fragment and the 3’-extremity of the last fragment were flanked respectively by the human cytomegalovirus promoter (pCMV) and the hepatitis delta virus ribozyme followed by the simian virus 40 polyadenylation signal (HDR/SV40pA). As previously described, PCR products were purified and treated by a digestion step with the restriction enzyme *Dpn*I (New England Biolabs) to ensure complete removal of the infectious clone used as template [10].

### Cell transfection

Cell transfection was performed as previously described [11]. Briefly, a total amount of 1 μg of DNA (infectious clone or equimolar mix of the three DNA fragments) were transfected into a 12.5 cm^2^ culture flask of subconfluent HEK-293 cells. Each virus was then passaged twice using Vero cells. Clarified cell supernatants (virus stocks) were used to perform vector competence study and whole-genome sequencing.

### Vector competence study

Five to seven day-old females were fed during 30 min with an infectious blood meal containing 2 ml of washed rabbit erythrocytes and 1 ml of viral suspension supplemented with a phagostimulant (ATP) at a final concentration of 5 mM. The final infectious titer in blood meals was 10^6.8^ TCID50/mL. Non-engorged females were then discarded. Engorged females were transferred in cardboard containers and maintained at 28°C with 10% sucrose.

Batches of 30 mosquitoes were analyzed at days 6 and 9 post-infection for each condition described in **Fig 2**. Days post-infection were chosen based on kinetics of CHIKV dissemination and transmission efficiencies in *Ae*. *aegypti* and *Ae*. *albopictus* mosquitoes (**Fig B in S1 File**). To assess viral dissemination, mosquito heads were removed and ground in 250 μL of Leibovitz L15 medium (Life technologies) supplemented with 2% FBS. Clarified supernatant was used to perform a TCID_50_ assay. To assess viral transmission, saliva was collected from individual females. For saliva collection, wings and legs were removed and the proboscis was inserted into a 20 μL tip containing 5 μL of FBS. After 45 min of salivation, FBS containing saliva was transferred into 45 μL of Leibovitz for titration (TCID_50_ assay).

### Tissue-culture infectious dose 50 (TCID_50_) assay

96-well plates culture of confluent Vero cells were used. Serial dilutions of samples were tested as previously described [15]. The procedure varied depending of the nature of the sample tested.

#### 1/ Mosquito samples

Mosquito heads: each serial 10-fold dilution was tested four times (first dilution: 1/5). Mosquito saliva: because sample size is very small, each serial 5-fold dilution was tested once (first dilution: 1/2.5). Amphotericine B was added to cell medium (final concentration: 2.50 μg/mL; Life technologies). Serial dilutions of samples were removed 2 hours after infection and 150 μL of fresh medium (3% FBS) was added in each well.

#### 2/ Cell supernatants

Each serial 10-fold dilution was tested six times (first dilution: 1/10) as previously described [15].

In all cases, plates were incubated for 7 days and scored for absence or presence of cytopathic effect (CPE) in each well. Determination of infectious titres TCID_50_/ml was performed using the method of Reed and Muench [16].

### Complete genome sequencing

Viral RNA was extracted from 80 μL of clarified supernatants from grounded heads using the NucleoSpin 8 / 96 Virus kit (Macherey-Nagel). As previously described, set of specific primer pairs was used to generate overlapping amplicons covering the full length genome [10].

Sequencing was performed using the Ion PGM Sequencer [17] (Life Technologies). Read analyses were performed as previously described [18]. To assess the genetic diversity of viral populations, mutation frequency for each position was calculated as the number of mutated reads divided by the total number of reads at that site. Only substitution with a frequency ≥2% were taken into account for analysis (**Table A in S1 File**).

### Statistical analysis

All the tests were carried out with the R software [19]. This includes Shapiro-Wilk test, Wilcoxon test, Fischer test, and Student t-test.

## Supporting information

S1 File Figure A: Mutation distribution.
Figure B: kinetics of IC_virus dissemination and transmission efficiencies in *Aedes* mosquitoes.
Table A: List of the mutations detected in this study.
(PDF)
