# Supplementary material for: Comparison of chikungunya viruses generated using infectious clone or the Infectious Subgenomic Amplicons (ISA) method in Aedes mosquitoes
Source: PLoS One. 2018 Jun 28;13(6):e0199494. doi: 10.1371/journal.pone.0199494 (PMC6023229; doi:10.1371/journal.pone.0199494)
Supplement: S1 File — Figure A: Mutation distribution. Figure B: kinetics of IC_virus dissemination and transmission efficiencies in Aedes mosquitoes. Table A: List of the mutations detected in this study. (PDF) [file pone.0199494.s001.pdf]

Supporting Information S1 File

Figure A: Mutation distribution

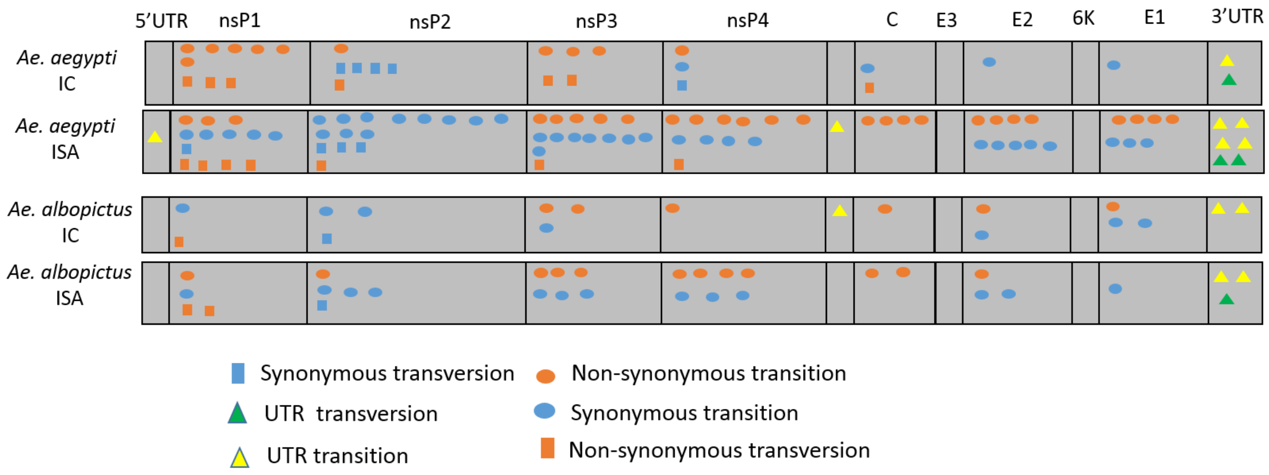

Figure B: kinetics of IC_virus dissemination and transmission efficiencies in *Aedes* mosquitoes

At days 2, 3, 6, 9, and 14 after an infectious blood meal, mosquitoes were sacrificed. Heads and saliva were collected for determination of dissemination (panel **A**) and transmission (panel **B**) efficiencies. Five heads were used to assess dissemination efficiencies. 27-30 saliva were used to assess transmission efficiencies except at day 14 post-infection with *Ae. albopictus* mosquitoes (n=5).

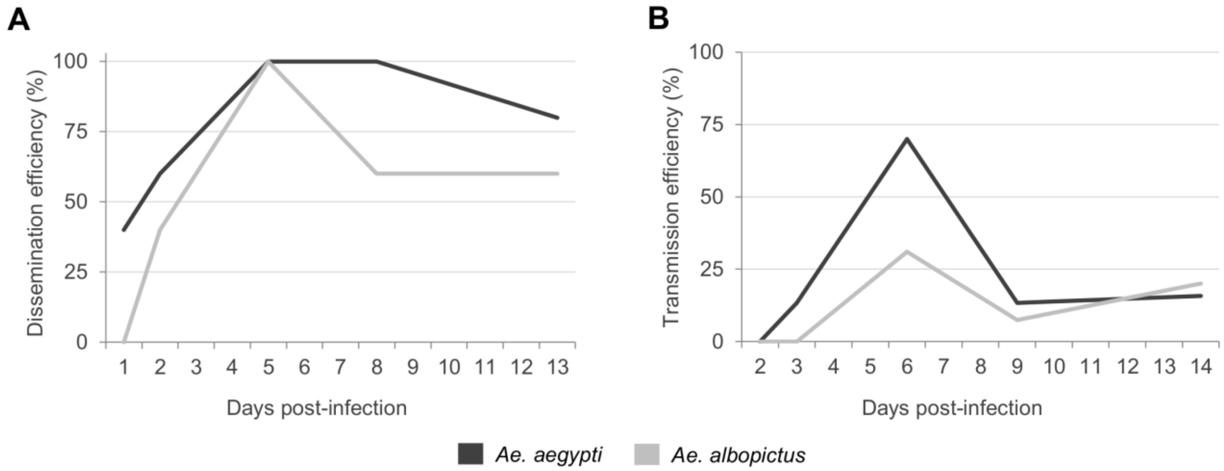

Table A: List of the mutations detected in this study

* : Mosquito heads (*Ae. Aegypti* or *albopictus*) ; Cell Sup. means cell supernatant (virus stock used to infect mosquitoes)

Nucleotide position	Dpi	Region	Nucleotide change	AA change	Substitution frequency	Sample*	Mosquito identification number	Reverse genetic method
25	J6	5'UTR	C→T	-	0,02	Ae. aegypti	19	ISA
502	J6	nsp1	A→G	-	0,03	Ae. aegypti	19	ISA
502	J6	nsp1	A→G	-	0,44	Ae. albopictus	28	ISA
502	-	nsp1	A→G	-	0,21	Cell Sup.	-	ISA
540	J6	nsp1	C→T	A-->V	0,04	Ae. aegypti	20	IC
546	J6	nsp1	A→T	H-->L	0,04	Ae. aegypti	20	IC
710	-	nsp1	T→C	-	0,03	Cell Sup.	-	ISA
836	J6	nsp1	C→T	-	0,99	Ae. aegypti	18	ISA
836	J6	nsp1	C→T	-	0,99	Ae. aegypti	17	ISA
856	J9	nsp1	G→A	-	0,15	Ae. aegypti	6	ISA
881	-	nsp1	A→C		0,03	Cell Sup.	-	ISA
1105	-	nsp1	C→A	-	0,03	Cell Sup.	-	ISA
1113	J6	nsp1	T→G	L-->R	0,02	Ae. aegypti	21	IC
1113	J6	nsp1	T→G	L-->R	0,02	Ae. aegypti	23	IC
1113	J6	nsp1	T→G	L-->R	0,02	Ae. aegypti	21	ISA
1113	J6	nsp1	T→G	L-->R	0,02	Ae. aegypti	20	ISA
1113	J6	nsp1	T→G	L-->R	0,03	Ae. aegypti	22	ISA
1247	J6	nsp1	T→C	F-->L	0,03	Ae. aegypti	20	IC
1247	J6	nsp1	T→C	F-->L	0,04	Ae. aegypti	21	IC
1247	J6	nsp1	T→C	F-->L	0,03	Ae. aegypti	23	IC
1247	J6	nsp1	T→C	F-->L	0,03	Ae. aegypti	24	IC
1247	J6	nsp1	T→C	F-->L	0,03	Ae. aegypti	25	IC
1247	J6	nsp1	T→C	F-->L	0,02	Ae. aegypti	21	ISA
1247	J6	nsp1	T→C	F-->L	0,02	Ae. aegypti	20	ISA
1247	J6	nsp1	T→C	F-->L	0,03	Ae. aegypti	18	ISA
1247	J6	nsp1	T→C	F-->L	0,02	Ae. aegypti	24	ISA
1247	J6	nsp1	T→C	F-->L	0,03	Ae. aegypti	17	ISA
1405	J6	nsp1	T→G	-	0,99	Ae. aegypti	21	ISA
1503	J6	nsp1	C→A	P-->Q	0,05	Ae. albopictus	4	ISA
1525	J6	nsp1	C→T	-	0,93	Ae. aegypti	22	ISA
1590	J6	nsp1	T→G	L-->R	0,03	Ae. aegypti	19	ISA
1590	J6	nsp1	T→G	L-->R	0,44	Ae. albopictus	28	ISA
1590	-	nsp1	T→G	L-->R	0,22	Cell Sup.	-	ISA
1659	J6	nsp1	A→G	Q-->R	0,99	Ae. albopictus	3	ISA
1711	J6	nsp2	C→T	-	0,97	Ae. aegypti	24	ISA

1966	J9	nsp2	A→C	K-->T	0,04	Ae. aegypti	17	IC
2188	J6	nsp2	A→C	-	0,02	Ae. aegypti	20	IC
2188	J6	nsp2	A→C	-	0,03	Ae. aegypti	21	IC
2188	J6	nsp2	A→C	-	0,02	Ae. aegypti	22	IC
2188	J6	nsp2	A→C	-	0,02	Ae. aegypti	23	IC
2188	J6	nsp2	A→C	-	0,02	Ae. albopictus	2	IC
2188	J6	nsp2	A→C	-	0,02	Ae. aegypti	18	ISA
2188	J6	nsp2	A→C	-	0,03	Ae. aegypti	17	ISA
2188	J6	nsp2	A→C	-	0,03	Ae. aegypti	22	ISA
2188	J6	nsp2	A→C	-	0,02	Ae. albopictus	28	ISA
2192	J6	nsp2	A→C	I-->L	0,02	Ae. aegypti	21	ISA
2239	J6	nsp2	A→T	-	0,03	Ae. aegypti	19	ISA
2346	J9	nsp3	T→C	M-->T	0,04	Ae. aegypti	18	IC
2458	J6	nsp2	T→C	-	0,03	Ae. aegypti	19	ISA
2458	J6	nsp2	T→C	-	0,99	Ae. aegypti	18	ISA
2458	J6	nsp2	T→C	-	0,99	Ae. aegypti	21	ISA
2458	J6	nsp2	T→C	-	0,99	Ae. aegypti	17	ISA
2458	J6	nsp2	T→C	-	0,03	Ae. albopictus	4	ISA
2458	J6	nsp2	T→C	-	0,07	Ae. albopictus	28	ISA
2458	-	nsp2	T→C		0,28	Cell Sup.	-	ISA
2592	J6	nsp2	T→C	V-->A	0,02	Ae. aegypti	20	IC
2743	J9	nsp2	C→T	-	0,97	Ae. aegypti	2	ISA
2890	J6	nsp2	T→C	-	0,12	Ae. albopictus	5	ISA
3068	J6	nsp2	A→G	S-->G	0,7	Ae. albopictus	27	ISA
3424	J6	nsp2	C→T	-	0,98	Ae. aegypti	21	ISA
4027	J6	nsp2	C→T	-	0,02	Ae. aegypti	20	ISA
4027	J6	nsp2	C→T	-	0,75	Ae. aegypti	19	ISA
4075	J6	nsp2	T→C	-	0,99	Ae. aegypti	18	ISA
4075	J6	nsp2	T→C	-	0,99	Ae. aegypti	17	ISA
4270	J6	nsp3	A→G	-	0,02	Ae. aegypti	19	ISA
4270	J6	nsp3	A→G	-	0,99	Ae. aegypti	23	ISA
4270	J9	nsp3	A→G	-	0,99	Ae. aegypti	5	ISA
4271	J6	nsp3	A→G	I-->V	0,02	Ae. albopictus	28	ISA
4339	J6	nsp3	C→T	-	0,99	Ae. aegypti	23	ISA
4495	J6	nsp3	C→T	-	0,02	Ae. albopictus	28	ISA
4639	J9	nsp3	A→G	-	0,99	Ae. aegypti	2	ISA
4639	J9	nsp3	A→G	-	0,99	Ae. aegypti	6	ISA
4639	J9	nsp3	A→G	-	0,99	Ae. aegypti	7	ISA
4639	J6	nsp3	A→G	-	0,02	Ae. albopictus	28	ISA
5243	J6	nsp3	T→C	S-->P	0,04	Ae. aegypti	18	ISA
5243	J6	nsp3	T→C	S-->P	0,99	Ae. aegypti	19	ISA

5243	J6	nsp3	T→C	S-->P	0,99	Ae. aegypti	21	ISA
5243	J6	nsp3	T→C	S-->P	0,99	Ae. aegypti	17	ISA
5243	J6	nsp3	T→C	S-->P	0,03	Ae. albopictus	4	ISA
5266	J6	nsp3	A→G	-	0,99	Ae. albopictus	3	ISA
5486	J6	nsp3	T→C	S-->P	0,03	Ae. aegypti	19	ISA
5486	J6	nsp3	T→C	S-->P	0,45	Ae. albopictus	28	ISA
5601	J9	nsp3	C→T	T-->I	0,02	Ae. aegypti	17	IC
5641	J6	nsp3	G→A	-	0,02	Ae. albopictus	28	ISA
5643	J6	nsp3	T→C	L-->S	0,79	Ae. aegypti	21	IC
5663	J6	nsp3	G→T	G-->W	0,02	Ae. aegypti	23	IC
5663	J6	nsp3	G→T	G-->W	0,02	Ae. aegypti	24	IC
5663	J6	nsp3	G→T	G-->W	0,02	Ae. aegypti	19	ISA
5933	J6	nsp4	T→A	S-->T	0,04	Ae. aegypti	21	ISA
5968	J6	nsp4	A→G	-	0,99	Ae. albopictus	3	ISA
6547	J6	nsp4	A→G	-	0,94	Ae. aegypti	17	ISA
6547	J6	nsp4	A→G	-	0,03	Ae. aegypti	19	ISA
6547	J6	nsp4	A→G	-	0,94	Ae. aegypti	18	ISA
6547	J6	nsp4	A→G	-	0,96	Ae. aegypti	21	ISA
6547	J6	nsp4	A→G	-	0,02	Ae. albopictus	4	ISA
6547	J6	nsp4	A→G	-	0,05	Ae. albopictus	28	ISA
6766	J9	nsp4	T→C	V-->A	0,06	Ae. aegypti	17	IC
6923	J6	nsp4	A→G	T-->A	0,88	Ae. aegypti	19	ISA
6923	J6	nsp4	A→G	T-->A	0,99	Ae. aegypti	20	ISA
6923	J6	nsp4	A→G	T-->A	0,99	Ae. aegypti	24	ISA
6923	J9	nsp4	A→G	T-->A	0,99	Ae. aegypti	1	ISA
6923	J9	nsp4	A→G	T-->A	0,99	Ae. aegypti	4	ISA
6923	J6	nsp4	A→G	T-->A	0,97	Ae. albopictus	4	ISA
6923	J6	nsp4	A→G	T-->A	0,99	Ae. albopictus	5	ISA
6923	J6	nsp4	A→G	T-->A	0,38	Ae. albopictus	27	ISA
6923	J6	nsp4	A→G	T-->A	0,39	Ae. albopictus	28	ISA
6923	-	nsp4	A→G	T-->A	0,23	Cell Sup.	-	ISA
7174	J6	nsp4	T→C	-	0,81	Ae. aegypti	21	IC
7490	J9	nsp4	G→T	-	0,99	Ae. aegypti	21	IC
7540	J9	J	C→T	-	0,99	Ae. aegypti	6	ISA
7628	J6	capside	C→T	P-->L	0,98	Ae. aegypti	17	ISA
7628	J6	capside	C→T	P-->L	0,04	Ae. aegypti	19	ISA
7628	J6	capside	C→T	P-->L	0,98	Ae. aegypti	21	ISA
7628	J6	capside	C→T	P-->V	0,98	Ae. aegypti	18	ISA
7628	J6	capside	C→T	P-->L	0,02	Ae. albopictus	4	ISA
7628	J6	capside	C→T	P-->L	0,08	Ae. albopictus	28	ISA
7628	-	capside	C→T	P-->L	0,28	Cell Sup.	-	ISA

8397	J6	E3	C→T	-	0,02	Ae. aegypti	23	IC
8612	-	E2	A→G	E-->G	0,02	Cell Sup.	-	ISA
8766	J6	E2	A→G	-	0,99	Ae. aegypti	17	ISA
8766	J6	E2	A→G	-	0,03	Ae. aegypti	19	ISA
8766	J6	E2	A→G	-	0,99	Ae. aegypti	18	ISA
8766	J6	E2	A→G	-	0,99	Ae. aegypti	21	ISA
8766	J6	E2	A→G	-	0,02	Ae. albopictus	4	ISA
8766	J6	E2	A→G	-	0,66	Ae. albopictus	28	ISA
8766	-	E2	A→G	-	0,15	Cell Sup.	-	ISA
9100	J9	E2	A→G	N-->D	0,99	Ae. aegypti	2	ISA
9100	J9	E2	A→G	N-->D	0,99	Ae. aegypti	6	ISA
9100	J9	E2	A→G	N-->D	0,99	Ae. aegypti	7	ISA
9100	J6	E2	A→G	N-->D	0,03	Ae. albopictus	28	ISA
9100	-	E2	A→G	N-->D	0,1	Cell Sup.	-	ISA
9360	J9	E2	C→T	-	0,07	Ae. aegypti	4	ISA
9505	J6	E2	C→T	P-->S	0,03	Ae. aegypti	24	ISA
9690	-	E2	T→C	-	0,02	Cell Sup.	-	ISA
9795	J9	E2	C→T	-	0,09	Ae. aegypti	17	IC
10217	J9	E1	A→G	D-->G	0,02	Ae. aegypti	2	ISA
10402	J6	E1	T→C	Y-->H	0,04	Ae. aegypti	23	ISA
10671	J6	E1	G→A	-	0,99	Ae. albopictus	14	IC
10719	J9	E1	T→C	-	0,98	Ae. aegypti	6	ISA
10719	J9	E1	T→C	-	0,99	Ae. aegypti	2	ISA
10719	J9	E1	T→C	-	0,99	Ae. aegypti	7	ISA
10719	J6	E1	T→C	-	0,02	Ae. albopictus	28	ISA
10761	J9	E1	A→G	-	0,07	Ae. aegypti	21	IC
10943	J6	E1	T→C	I-->T	0,02	Ae. aegypti	17	ISA
10943	J6	E1	T→C	I-->T	0,02	Ae. aegypti	18	ISA
11137	J9	E2	C→A	P-->T	0,02	Ae. aegypti	21	IC
11315	J6	3'UTR	T→C	-	0,12	Ae. albopictus	28	ISA
11329	J6	3'UTR	T→G	-	0,02	Ae. aegypti	21	ISA
11490	J6	3'UTR	T→A	-	0,06	Ae. albopictus	4	ISA
11550	J6	3'UTR	T→C	-	0,8	Ae. aegypti	21	IC
11557	J9	3'UTR	C→A	-	0,04	Ae. aegypti	21	IC
11557	J6	3'UTR	C→A	-	0,08	Ae. aegypti	17	ISA
11642	J9	3'UTR	T→C	-	0,99	Ae. aegypti	5	ISA
11644	J9	3'UTR	A→G	-	0,99	Ae. aegypti	2	ISA
11644	J9	3'UTR	A→G	-	0,99	Ae. aegypti	6	ISA
11644	J9	3'UTR	A→G	-	0,99	Ae. aegypti	7	ISA
11644	J6	3'UTR	A→G	-	0,02	Ae. albopictus	28	ISA
11644	-	3'UTR	A→G	-	0,1	Cell Sup.	-	ISA
